# MRI T1 Contrast-Enhanced Signal Intensity Is a Prognostic Indicator of Imatinib Therapy in Desmoid-Type Fibromatosis

**DOI:** 10.3389/fonc.2021.615986

**Published:** 2021-03-15

**Authors:** Hui Ci Zhu, Shi Xing Xu, Xiao Ting Li, Zhen Guan, Shu Li, Ying-Shi Sun

**Affiliations:** ^1^ Department Radiology, Peking University Cancer Hospital, Beijing, China; ^2^ Department Plastic Surgery No.6, Plastic Surgery Hospital, Beijing, China; ^3^ Department Bone and Soft Tissue Tumors, Peking University Cancer Hospital, Beijing, China

**Keywords:** aggressive fibromatosis, systemic therapy, imatinib, response evaluation criterion in solid tumors (RECIST), magnetic resonance imaging

## Abstract

**Objective:**

To investigate the efficiency of pre-therapy magnetic resonance imaging (MRI) features in predicting the prognosis of desmoid-type fibromatosis patients treated with imatinib.

**Materials and Methods:**

A total of 38 desmoid-type fibromatosis patients treated with imatinib were collected in this retrospective study. The high signal intensity on pre-therapy MRI was evaluated on axial T2 and T1 contrast-enhanced sequences with fat suppression. Cox regression and Kaplan–Meier analyses explored the correlation between clinical or radiographic characteristics and progression-free survival (PFS).

**Results:**

Hyperintense T1 contrast enhancement (CE) proportion (≥ 75%) was identified as an independent predictor for PFS. Patients with hyperintense T1 CE proportion <75% demonstrated no progression, while patients with hyperintense T1 CE proportion ≥75% demonstrated a progression rate of 78.4%.

**Conclusion:**

Hyperintense T1 CE proportion in the tumor is a potential predictor of disease progression in patients with desmoid-type fibromatosis treated with imatinib. Hyperintense T1 CE proportion <75% indicates progression-free during treatment.

## Introduction

Desmoid-type fibromatosis (DF) is a locally aggressive fibroblastic proliferation without metastatic potential (1, 2). The incidence of DF is 2–4/million individuals and accounts for 0.03% of all neoplasms (2). Patients may be asymptomatic or present severe loss of organ function and significant morbidity (3, 4). Some tumors may progress rapidly, while others can remain stable for a prolonged period (1, 5, 6). Primarily, the treatment lessens functional impairment.

Interestingly, the treatment method of DF has changed dramatically over the past decade. Because of the wide range (from 25 percentage to 70 percentage) and a high incidence of local recurrence, a subset cannot be subjected to complete excision. In this case, clinicians accept non-surgical approaches at the initiation of management, including observation, systemic therapies, radiation therapy, and radiofrequency ablation (1, 7–10). Chemotherapy with conventional or targeted agents is a major strategy for the treatment of DF, especially for unresectable lesions that are adjacent to vital organs. Although several potential therapies are available, there is still a lack of evidence to guide the clinicians to choose the optimal therapy for specific patients (1, 8)

Imatinib is one of the most widely used tyrosine kinase inhibitors (TKIs) that is effective in progressive DF therapy (11–13). However, the overall response rate (6–100%) differed significantly from that reported previously (14).

The role of magnetic resonance imaging (MRI) is widely recognized in DF during diagnosis and follow-up (1, 3, 15, 16). MRI signal intensity may reveal the proportion of collagen fibers, myxoid matrix, spindle cells, and cellular stroma (3, 17–19). Some studies have proved the effectiveness of MRI signal in managing the treatment decision for DF patients (17, 19, 20), while recent studies found that DF growth was associated with T2 hyperintensity (21). We hypothesized that the MRI features, including shape, infiltrative margin, hyperintensity, and adjacent structure involvement, may be used as imaging biomarkers that are associated with the prognosis of imatinib-treated DF.

Thus, the objective of this retrospective cohort study was to investigate the effectiveness of pre-therapy MRI features in predicting the prognosis of DF patients treated with imatinib.

## Materials and Methods

This retrospective, non-interventional study was approved by our institutional review board, with a waiver of informed consent.

### Study Population

**Figure 1:**
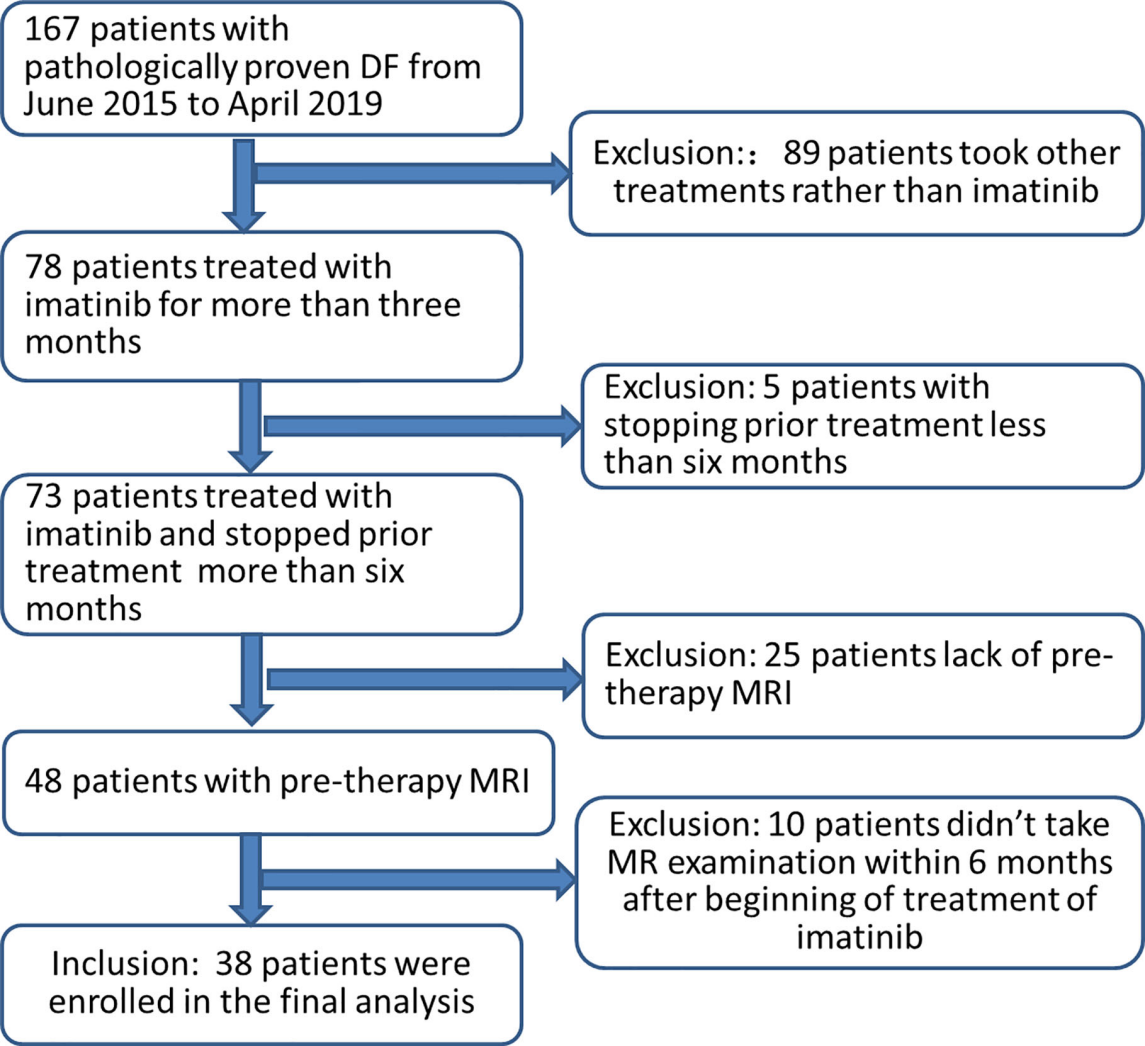
Schematic of patient recruitment.

Patients with histologically documented DF from the database in our hospital from June 2015 to April 2019 were assimilated for this study. Tumors were measurable according to RECIST 1.1 criteria using MRI (22). The inclusion criteria were as follows: (a) patients treated with imatinib for >3 months, and no concomitant treatment was administered; (b) availability of pre-therapy MRI including T2-weighted images (T2WI) and T1 contrast enhancement (CE) images; (c) availability of MRI follow-up images every 3–6 months after the treatment. The exclusion criteria were as follows: (a) the use of treatments other than imatinib, (b) prior treatment ended <6 months ago, (c) lack of pre-therapy MRI, and (d) patients did not undergo MR examination within 6 months after beginning treatment with imatinib. Finally, 38 patients were enrolled in the current analysis (**Figure 1**).

Clinical data were obtained from the electronic medical record, including gender, age, treatment history, tumor type (primary or recurrence), the number of follow-up MRI examinations, if the imatinib dose was adjusted and if there were changes in the treatment strategies.

The imatinib treatment consisted of a dose of 400 mg/day administered for all patients. The median duration of the treatment was 11.3 (range 3–36) months. Dose escalation, up to 600 mg/day, was observed in 19 (50.0%) cases. Subsequently, 16/38 patients changed the treatment strategy due to RECIST-defined disease progression under imatinib, while 22 patients continued imatinib treatment during follow-up. None of the patients suffered from intolerant side effects.

### Magnetic Resonance Imaging Protocol and Image Analysis

MRI examinations were performed on a 1.5T MRI scanner (Siemens Magnetom Aera, Germany) with a dedicated 18 channel phased array coil. The imaging protocol consisted of the following sequences (**Table 1**): coronal or sagittal turbo spin-echo (TSE) T2WI, axial TSE T1-weighted imaging (T1WI), axial TSE T2WI and fat-suppressed TSE T2WI, axial DIXON VIBE T1WI and T1WI CE imaging, and coronal or sagittal DIXON VIBE T1WI CE imaging. The axial fat-suppressed TSE T2WI parameters were as follows: repetition time (TR) 5,000–5,560 ms; echo time (TE), 74–85 ms; average, 1-2; bandwidth, 279–422 Hz/pixel; turbo factor, 14–16; slice thickness, 5–7 mm; gap 1–1.4 mm; matrix, 320× 256–320 × 288; field of view (FOV), 200–360 mm. The axial DIXON VIBE T1 CE imaging parameters were as follows: TR 6.7–6.84 ms; TE, 2.39 ms; flip angle, 10°; average, 2; bandwidth, 600 Hz/pixel; slice thickness, 4–5 mm; matrix, 288× 259–320 × 288; FOV, 260–360 mm. DIXON VIBE T1 CE sequence was obtained at 2 min after the injection. A 0.02 mmol/kg dose of gadopentetate dimeglumine (Magnevist; Bayer Healthcare) was injected in a peripheral vein at a rate of 2 ml/s using an injection pump. The acquisition protocols were adapted according to patient anatomy and tumor location.

**Table 1:** MRI parameters used in this study on a 1.5 T clinical scanner.

MR Sequence	Imaging plane	FOV^a^ (mm)	Matrix	Slice thickness/gap (mm)	TR/TE^b^ (ms)	Turbo factor	Number of signals averaged	Bandwidth (Hz/voxel)
T1	Axial	200–360	384×320-384×384	5–7/1–1.4	450–543/5.5-6.3	3	2	303–395
T2	Axial/Sagittal/Coronal	200–360	384×384	5–7/1–1.4	4000–6120/76-84	16–20	1-2	303–353
T2 FS^c^	Axial	200–360	320×256-320×288	5–7/1–1.4	5000–5560/74-85	14–16	1-2	279–422
T1 FS CE^d^	Axial/Sagittal/Coronal	260–360	288×259-320×288	4-5/0	6.7–6.84/2.39	NA	2	600

^a)^FOV, field of view; ^b)^TR/TE, repetition time/echo time; ^c)^FS, fat-suppressed; ^d)^CE, contrast enhancement.

MR images were extracted and assessed on a picture archiving and communication system (PACS) workstation (Carestream Health v.12.0; USA). The longest dimension of the tumor at pre-therapy and follow-up imaging were measured in any plane on T2WI images or T1 CE images. The peritumoral edema was not considered as a part of the tumor diameter, defined as a high fluid-like signal at T2WI with infiltrative and feathery borders that are distinguishable from the apparent tumor borders and without mass effects (23). All the observers were blinded to the treatment regimen and follow-up data; all the images for a patient were viewed concurrently. The following data were obtained from the pre-therapy MRI by two readers (ZHC and GZ with 8 and 3 years of experience of interpreting MR images, respectively) independently: location, compartment of origin (defined as subcutaneous, superficial fascial, intramuscular, and deep fascial/intermuscular) (17), multifocality (a lesion was considered multifocal if at least 1 cm of definitive normal tissue was detected on all the available sequences), dominant shape (round/oval, lobulated, and infiltrative), and adjacent structure involvement including neurovascular bundle and bone (none, adjacent, encased, and invaded). Next, the largest lesion of multifocal tumors was measured. If there was a discrepancy between the two radiologists, a third experienced radiologist (SYS, with 20 years of experience in interpreting MRI images) conducted the arbitration.

**Figure 2:**
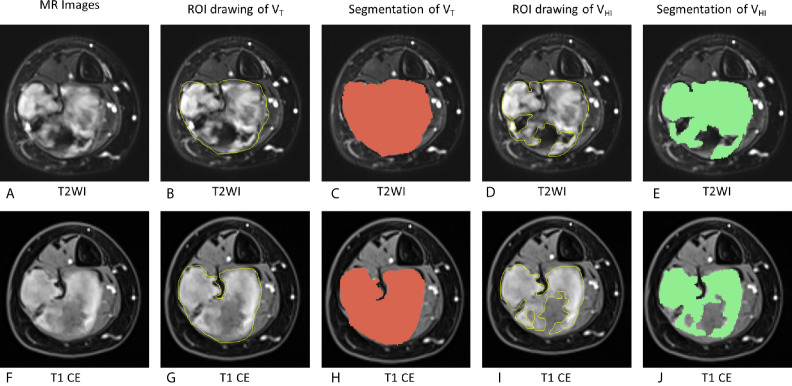
Schematic of evaluation of MRI hyperintensity of pre-therapy MRI. On T2WI, V_T_ = 162,695 mm^3^, V_HI_ = 100,871 mm^3^, HI = 62.0% (grade = 2). On T1 CE, V_T_ = 161,577 mm^3^, V_HI_ = 103,267 mm^3^, HI = 63.9% (grade = 2). V_T_, The tumor volume; V_HI_, volume of signal hyperintensity; HI, the hyperintensity percentage of the tumor. **(A–E)**, T2WI images. **(F–J)**: T1 CE images. **(A, F)**: MR images without any drawing. **(B, G)**: ROI drawing of the tumor volume (V_T_). **(C, H)**: Segmentation of V_T_. **(D, I)**: ROI drawing of the volume of signal hyperintensity (V_HI_). **(E, J)**: segmentation of V_HI_.

To evaluate the intensity of pre-therapy MRI, two radiologists (ZHC and GZ) manually drew the whole tumor and high signal intensity areas on each slice of axial T2 fat suppression image and T1 CE images (**Figure 2**). The areas were regarded as hyperintense if they were brighter than the adjacent muscles on both T2WI and T1CE sequences with fat suppression on the same image. The tumor volume (V_T_ in the following equation) and volume of signal hyperintensity (V_HI_ in the following equation) was calculated based on the sum of the areas on the images multiplied by the slice thickness using Slicer 4.8.1 software (Brigham and Women’s Hospital, Boston, MA, USA and Massachusetts Institute of Technology, Cambridge, MA, USA). The hyperintensity percentage of tumor (HI in the following equation) was determined according to the following formula:

$$\text{HI}=\frac{V_{HI}}{V_{T}}\times 100\%$$

HI of each lesion on T2WI images and T1 CE images was graded as follows (19, 24, 25):

grade 0:HI=0-10%

grade 1:HI=11-50%

grade 2:HI=51-74%

grade 3:HI=75-100%

This hyperintensity was estimated before the measurements of the longest dimension of the tumor on pre-therapy MRI. Next, we analyzed the associations of the hyperintensity of the tumor according to the progression-free survival (PFS).

### Statistical Analysis

The primary endpoint was a progression for lesions using RECIST1.1 criteria (22). Tumors that decreased at least 30% in diameter were considered to be in regression, while tumors that increased by at least 20% in diameter were considered to be progressing; otherwise, the lesion was considered stable. PFS was calculated from the date of pre-therapy MRI to the date of radiographic progression or the date of the last follow-up MRI.

Kaplan–Meier plot with log-rank test was conducted to compare the survival curves. Univariate Cox analysis was used to calculate the hazard ratio (HR) with 95% confidence interval (CI). The correlation between clinical characteristics and MRI signals was detected using chi-square test or Fisher’s exact test.

The interobserver agreement was evaluated by calculating the intraclass correlation coefficient (ICC): 0–0.40, 0.41–0.75, >0.75 indicated poor, moderate, and good agreement, respectively. If good agreement was achieved, HI was recorded by taking an average of the two radiologists’ data.

## Results

### Characteristics of the Study Population

Patient characteristics and image characteristics are summarized in **Tables 2** and **3**, respectively. The mean age of the cohort was 29 (range 14–47) years. The tumor was multifocal in 10/38 cases (26.3%), only one DF lesion was observed in the remaining 28 (73.3%) patients. The dominant shape of the tumor was round/oval in 7 cases (18.4%), lobulated in 14 cases (36.8%), and infiltrative in 17 cases (44.8%). Moreover, 10 (26.3%) cases did not show any adjacent structure involvement, while 28 (73.7%) cases showed various degrees of adjacent structure involvement. The mean tumor size was 80.7 ± 54.2 mm.

**Table 2:** Patient characteristics.

	Mean ± std/No. (percentage)
Age at diagnosis (years)	29.0 ± 9.1
Sex	
Male	12 (31.6%)
Female	26 (68.4%)
History of treatment	
Primary	7 (18.4%)
Recurrence with previous surgery	17 (44.7%)
Recurrence with previous systemic therapy	10 (26.3%)
Recurrence with previous surgery and radiation/RFA	4 (10.5%)
Location	
Upper or lower extremity	17 (44.7%)
Chest or abdominal wall	5 (13.2%)
Torso (including head and neck)	15 (39.5)
Intra-abdominal	1 (2.6%)

RFA, radiofrequency ablation; systemic therapy, low-dose chemotherapy, anti-inflammatory, or endocrine therapy.

**Table 3:** Imaging characteristics.

	Mean ± std/No. (percentage)
Dominant shape	
Round/oval	7 (18.4%)
Lobulated	14 (36.8%)
Infiltrative	17 (44.8%)
Multifocality	
No	28 (73.7%)
Yes	10 (26.3%)
Adjacent structure involvement	
None	10 (26.3%)
Adjacent	9 (23.7%)
Encased	14 (36.8%)
Invaded	5 (13.2%)
Compartment of origin	
Subcutaneous	1 (2.6%)
Superficial fascial	2 (5.3%)
Intramuscular	8 (21.1%)
Deep fascial/intermuscular	27 (71.0%)

All 38 patients underwent 102 MR examinations. The mean follow-up interval was 4.6 months, and the median for MRI follow-up was 3 (range: 1–7) months. Their median follow-up was 23.1 months (range 3 to 36). At the end of the follow-up, RECIST1.1-based response was as follows: none of the patients achieved complete response (CR) or partial response (PR); 22 (57.9%) patients exhibited stable disease (SD); 16 (42.1%) patients presented progressive disease (PD).

### Prognostic Factors According to Progression

The ICC inter-reader reliability and variability in the evaluation of MRI signal hyperintensity was 0.815 (95% CI: 0.748–0.853) and 0.885 (95% CI: 0.814–0.967) for T2 and T1 CE imaging, respectively.

**Figure 3:**
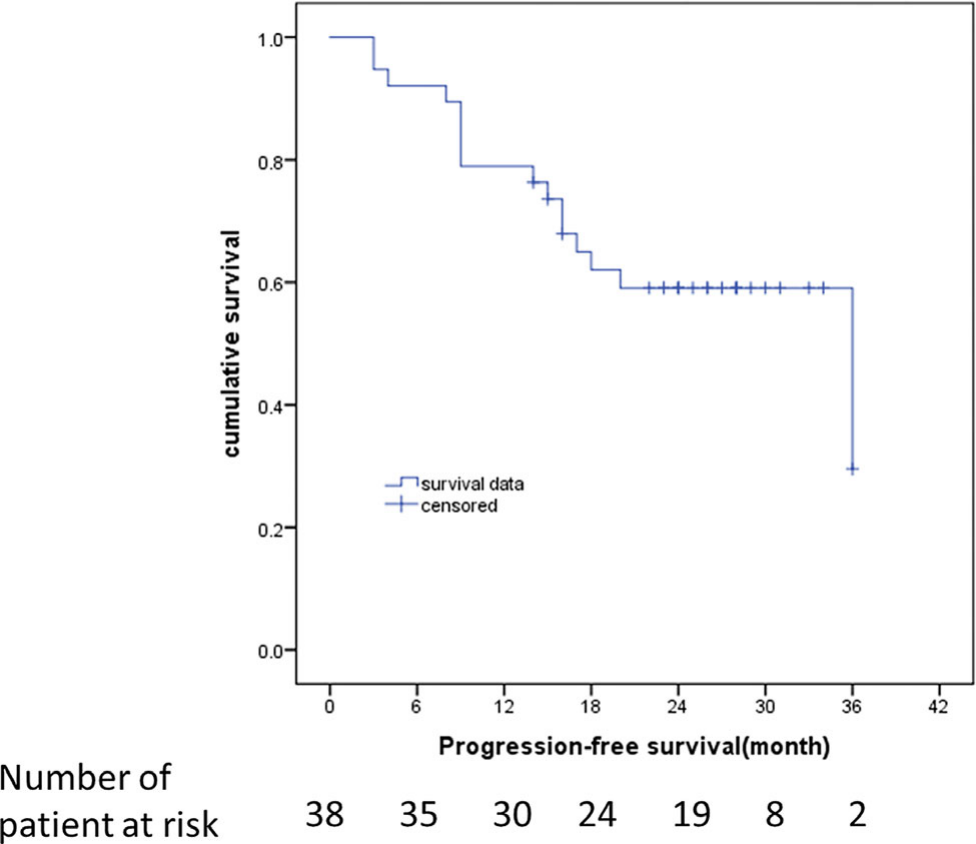
Kaplan–Meier estimates of the duration of PFS at the time of last assessment. RECIST 1.1 was used to identify disease progression. Data from patients who did not have progression were censored and marked by a tick.

**Figure 4:**
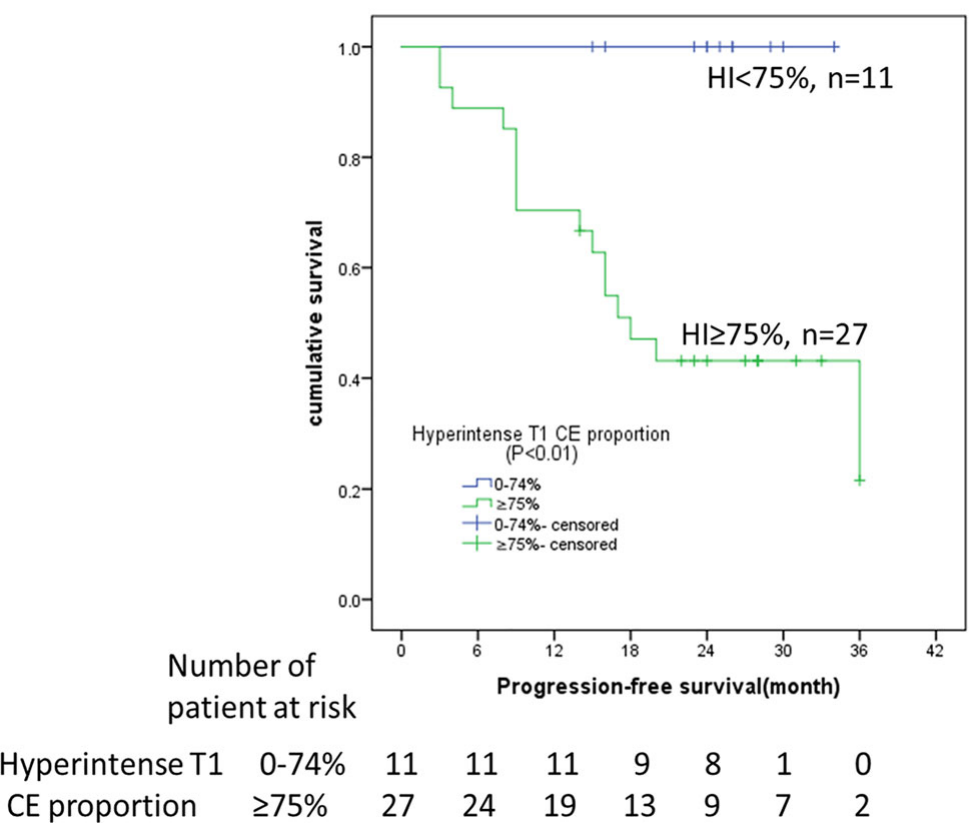
Kaplan–Meier analyses of PFS in 38 DF patients with imatinib treatment. Patients stratified by hyperintense T1 CE proportion (<75 *vs*. ≥75%) on pre-therapy MRI. HI, hyperintense T1 CE proportion; CE, contrast enhancement.

**Figure 5:**
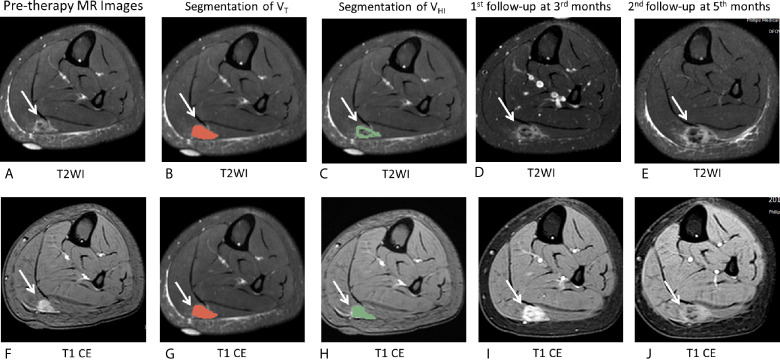
MR images of the representative case. **(A–J)**, A 16-year-old female patient with DF of right calf muscle with imatinib treatment (arrows). Pre-therapy MR images refer to examination before imatinib treatment. Pre-therapy **(A, F)** diameter, 20.0 mm; On T2WI, V_T_ = 6,242 mm^3^, V_HI_ = 6,242 mm^3^, HI = 100% (grade = 3); On T1 CE, V_T_ = 6,357 mm^3^, V_HI_ = 6,357 mm^3^, HI = 100% (grade = 3). Segmentation of V_T_ (**B, G**, red zone) and segmentation of V_HI_ (**C, H**, green zone). 1^st^ follow-up at 3 months after imatinib treatment **(D, I)** Diameter, 23.5 mm indicates a 17.5% increase in tumor size as well as a decrease in T2 and T1 CE signal intensity. The tumor response is SD, according to RECIST1.1. Second follow-up at 6 months since imatinib treatment **(E, J)** diameter, 48.0 mm shows a 240% increase in tumor size as well as a decrease in T2 and T1 CE signal intensity. The tumor response assessment is PD, according to RECIST1.1. After the second follow-up examination, this patient changed treatment strategy to chemotherapy.

**Figure 6:**
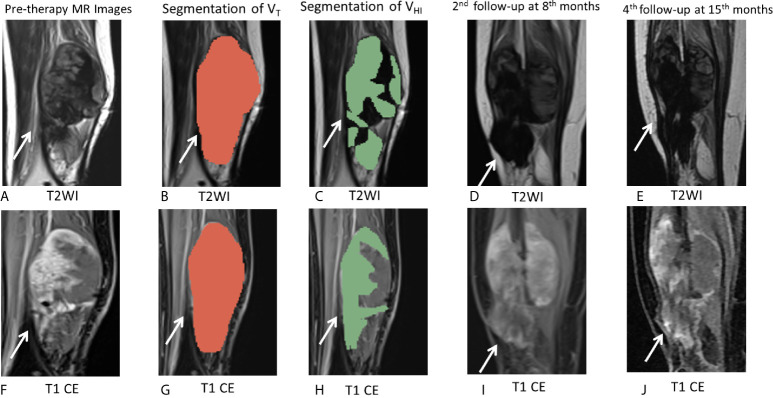
MR images of the representative cases. **(A–J)** A 26-year-old female patient with DF of right calf muscle with imatinib treatment of (arrows). Pre-therapy MR images refer to examination before imatinib treatment. Pre-therapy **(A, F)** diameter, 113.0 mm; On T2WI, V_T_ = 162,695 mm^3^, V_HI_ = 97,617 mm^3^, HI = 60% (grade = 2); On T1 CE, V_T_ = 163,750 mm^3^, V_HI_ = 106,438 mm^3^, HI = 65% (grade = 2). Segmentation of V_T_ (**B, G**, red zone) and segmentation of V_HI_ (**C, H**, green zone). Second follow-up at 8 months since imatinib treatment **(D, I)** diameter, 112.0 mm indicate a 0.9% decrease in tumor size. The tumor response assessment is SD, according to RECIST1.1. Fourth follow-up at 15 months since imatinib treatment **(E, J)** diameter, 105.0 mm indicate a 7.1% decrease in tumor size as well as a decrease in T2 and T1 CE signal intensity. The tumor response assessment is SD, according to RECIST1.1. During 15 months of follow-up during imatinib treatment, there was no evidence of progression.

The median follow-up was 23 months (95% CI: 16-26 months). The survival curves for PFS are presented in **Figure 3**. None of the patients (n = 11) with hyperintense T1 CE proportion 0–74% suffered death or progression, and none of the patients (n = 27) with hyperintense T1 CE proportion >75% were deceased; therefore, the overall survival rate was 100%. The median time to progression was 18 months for patients with hyperintense T1 CE proportion >75%. The PFS rates for these patients at 6, 12, 18, 24, 30, and 36 months were 88.9, 70.4, 47.1, 43.1, 43.1, and 21.6%, respectively. Univariate analysis showed that only hyperintense T1 CE proportion was a significant predictor of PFS (P = 0.02) (**Table 4**, **Figure 4**). Two representative cases are presented in **Figures 5** and **6**, respectively.

**Table 4:** Univariate analysis of clinical and radiographic characteristics for PFS.

Characteristics		N	24^th^ month PFS rate (%)	HR	95% CI	P
Hyperintense T2 proportion	0–74%	6	83.3	1		0.27
	≥75%	32	55.5	1.42	0.76–2.65	
Hyperintense T1 CE proportion	0-74%	11	100	1		0.02*
	≥75%	27	43.1	3.37	1.23–9.25	
Size (mm)	1–49	11	80.0	1		0.15
	≥50	27	51.0	3.0	0.67–13.27	
Tumor volume(mm^3^)	1–96590	18	69.9	1		0.27
	≥96590	20	50.0	37	0.63–5.35	
Age(year)	1–29	18	71.4	1		0.13
	≥29	20	47.7	2.31	0.79–6.81	
Sex	Male	12	90.9	1		0.05
	Female	26	45.1	4.35	0.98–19.20	
Dominant shape	Round/oval	7	42.9	1		0.60
	Lobulated	14	70.7	0.51	0.13–2.03	0.34
	Infiltrative	17	57.0	0.83	0.24–2.84	0.77
Multifocality	No	28	58.6	1		0.87
	Yes	10	60.0	0.91	0.29–2.87	
Adjacent structure involvement	None	10	46.7	1		0.86
	Adjacent	9	66.7	0.81	0.22–3.01	0.75
	Encased	14	55.0	0.97	0.29–3.25	0.96
	Invaded	5	80.0			
Compartment of origin	Deep fascial/intermuscular	27	62.0	1		
	Intramuscular	8	72.9	1.00	0.32–3.16	
	Superficial fascial	2	0	1.00	0.06–12.32	
	Subcutaneous	1	0	/		

*P < 0.05; CI, confidence interval; HR, hazard ratio.

However, other variables, including hyperintense T2 proportion, size, tumor volume, age, gender, dominant shape, multifocality, adjacent structure involvement, and compartment of origin, were not significant for PFS (all P >0.05).

**Table 5** shows that patients with hyperintense T1 CE proportion ≥75% are >50-years-old (P = 0.04). Other clinical characteristics, including size, tumor volume, gender, dominant shape, multifocality, adjacent structure involvement, dose-escalation, and history of treatment, were not statistically significant (all P >0.05).

**Table 5:** Characteristics of the 38 DF patients with imatinib treatment stratified by HI proportion.

		HI on T1 CE <75% (n = 11)	HI on T1 CE≥75%(n = 27)	P
Size (mm)	1–49	4	6	0.43
	≥50	7	21	
Tumor volume (mm^3^)	1–96,590 ≥96,590	6 5	12 15	0.57
Age (years)	1–29	8	10	0.04*
	≥50	3	17	
Sex	Male	5	7	0.27
	Female	6	20	
Dominant shape	Round/oval	1	6	0.09
	Lobulated	7	7	
	Infiltrative	3	14	
Multifocality	No	8	20	1.00
	Yes	3	7	
Adjacent structure involvement	None	1	9	0.13
	Adjacent	4	5	
	Encased	3	11	
	Invaded	3	2	
Compartment of origin	Deep fascial/intermuscular	7	20	0.69
	Intramuscular	3	5	
	Superficial fascial	1	1	
	Subcutaneous	0	1	
Dose escalation	No	6	13	0.72
	Yes	5	14	
History of treatment	Primary	2	5	0.23
	Recurrence with previous surgery	7	10	
	Recurrence with previous medical therapy	2	8	
	Recurrence with previous surgery and radiation/RFA	0	4	

*P < 0.05; HI, hyperintense proportion; CE, contrast enhancement.

## Discussion

In this study, we identified whether pre-therapy MRI signal intensity is a reliable prognostic predictor of the progression of DF in patients treated with imatinib. Although a few previous studies have reported that some clinical characteristics may be used as prognostic indicators in DF patients (17, 25–27), this is the first study investigating the role of MRI in assessing the clinical outcome in DF patients treated with imatinib. The results indicated that hyperintense T1 CE proportion (≥75%) of pre-therapy MRI was strongly associated with RECIST-defined disease progression during the treatment with imatinib.

Although in small prospective studies, imatinib has been shown to have progression arrest activity (10–13) during the treatment of DF, beneficial prognostic biomarkers have not been identified. In the current study, the PFS at 6, 12, 18, and 24 months was 92.1, 78.9, 63.2, and 50.0%, respectively, which was in accordance with the results reported previously (14). We also found that hyperintense T1 CE proportion may be used as a novel and reliable imaging biomarker that can aid clinicians in selecting optimal treatment strategies in the future. In this study, all the 11 cases with <75% hyperintense T1 CE proportion presented during pre-therapy MRI showed a stable tumor response. Briefly, if <75% hyperintense T1 CE proportion was presented on pre-therapy MRI, this cohort of patients could be considered suitable for imatinib treatment, which might induce a high possibility of disease progression arrest. However, if ≥75% hyperintense T1 CE proportion was presented on pre-therapy MRI, more than half (59.3% in our study) of these patients might exhibit disease progression. Therefore, these findings support the idea that this group of patients can follow different treatment strategies, such as sorafenib, pazopanib, chemotherapy regimens, or radiotherapy (4, 14, 28).

**Figure 7:**
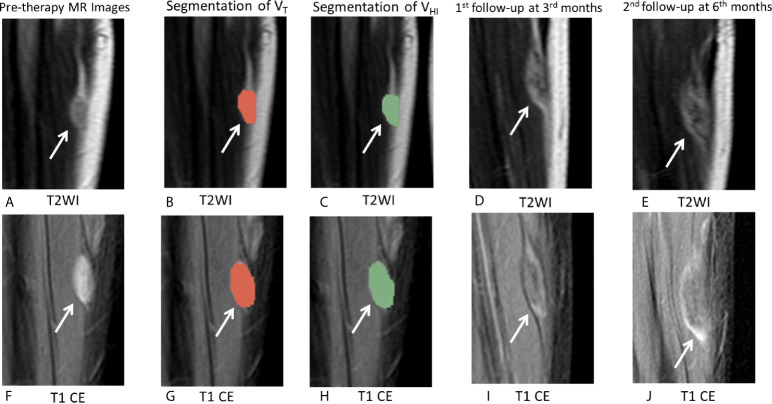
MRI images in a 18-year-old male patient with DF of left calf muscle with imatinib treatment (arrows). Pre-therapy **(A, F)** diameter, 21.0 mm; On T2WI, V_T_ = 5,477 mm^3^, V_HI_ = 418 mm^3^, HI = 7.5% (grade = 0); On T1 CE, V_T_ = 5,238 mm^3^, V_HI_ = 5,238 mm^3^, HI = 100% (grade = 3). Segmentation of V_T_ (**B, G**, red zonex) and segmentation of V_HI_ (**C, H**, green zone). First follow-up at 3 months since treatment with imatinib **(D, I)** diameter, 25.0 mm indicates a 19.0% increase in tumor size. The tumor response assessment is SD, according to RECIST1.1. Second follow-up at 5 months since treatment with imatinib **(E, J)** diameter, 36.0 mm indicates a 71.4% increase in tumor size. The tumor response assessment is PD, according to RECIST1.1.

For DF, signal intensity of MRI reflects the proportion of the neoplasms (3). An increase in collagenization in the tumor causes a decrease in T2 signal during treatment (15, 19, 29). The recent studies (4, 28) indicated that RECIST may underestimate the efficacy of DF treatment and that MRI signal intensity may be a better criterion. Michael (25) evaluated the tumor volume proportion showing hyperintense T2 signal at pre-therapy MRI and analyzed its associations with PFS during active observation. The study also demonstrated an association between tumor growth and hyperintense T2 signal (>90%). Inconsistent with the previous study, our results demonstrated that hyperintense T1 CE proportion was the only significant factor for PFS. Typically, high signal intensity in T2 and T1 CE reflects the different characteristics of the tumor. Histologically, increased signal intensity on T2WI may result from decreased collagen deposition and increased cellularity/myxoid matrix/cellular stroma. Increased signal intensity on T1 CE might result from increased cellularity and angiogenesis (3, 18, 30), which was in contrast to the hyperintensity in T2 and T1 CE (**Figure 7**).

The current study used a hierarchical approach to evaluate MRI signal intensity on pre-therapy images, which was supported by previous literature (19, 24). Two radiologists delineated the whole tumor areas and high signal intensity area by drawing on each slice to improve the accuracy of evaluation of signal hyperintensity on MRI. In order to reduce the variation between observers, we adopted a semiquantitative grading system. Although drawing the area of high signal intensity was subjective and complex, the consistency between the observers was good (ICC = 0.815 and 0.855), and the average time of drawing the region of interest (ROI) was acceptable (6–10 min). Hence, this grading system could be applied for the evaluation of MRI signal intensity of DF in clinical practice.

In contrast to previous efforts at deriving prognostic factors from clinical variables (17, 26, 31), other characteristics and MRI features, including size, age, gender, dominant shape, multifocality, adjacent structure involvement, and compartment of origin, were not associated with PFS. Since our study participants were patients treated with imatinib, it might introduce a selection bias, and hence, the findings cannot invalidate the results of the previous study, which included a larger number of patients.

Nevertheless, the present study has some limitations. First, this was a retrospective, single-center study with a small sample size and short follow-up period. But the pilot study defined a group of patients treated with imatinib. The prognostic value of hyperintense T1 CE proportion of pre-therapy MRI needs to be validated in a wider population with a prolonged follow-up period. Second, DF patients treated with other therapies, such as low-dose chemotherapy, endocrine therapy, other target therapy, and radiotherapy, should be incorporated into future studies to analyze the association between pre-therapy MRI signal intensity and prognosis. Third, since not all patients underwent genetic testing and inquiry of health-related quality of life, clinical and molecular findings, such as symptoms and expression of the *β-catenin* gene were not evaluated in this study. Thus, in the future, larger and prospective multicentric studies would substantiate these findings.

In conclusion, this study proved the role of hyperintense T1 CE proportion of pre-therapy MRI in predicting progression in DF patients treated with imatinib. Patients with tumors of hyperintense T1 CE proportion <75% have better PFS during imatinib treatment than those with >75%. Thus, we recommend MR as an imaging biomarker to assess the prognostic outcomes of imatinib-treated DF.

## Data Availability Statement

The original contributions presented in the study are included in the article/supplementary material. Further inquiries can be directed to the corresponding author.

## Author Contributions

HCZ and Y-SS designed the study. HCZ and SL provided the study materials or patients. HCZ, SXX, XTL, ZG, and Y-SS processed, analyzed, and interpreted the data. All authors wrote and edited the manuscript. All authors contributed to the article and approved the submitted version.

## Funding

This study was funded by National Key Research and Development Plan (2019YFC0117705, 2017YFC1309100), the Beijing Municipal Administration of Hospitals Clinical Medicine Development of Special Funding Support (No. ZYLX201803), 2019 SKY Imaging Research Fund of the Chinese International Medical Foundation(Project No.Z-2014-07-1912) and Beijing Hospitals Authority’Ascent Plan (Code:20191103).

## Conflict of Interest

The authors declare that the research was conducted in the absence of any commercial or financial relationships that could be construed as a potential conflict of interest.

## Abbreviations

DF, desmoid-type fibromatosis; PFS, progression-free survival; MRI, magnetic resonance imaging; CE, contrast enhancement; RECIST, response evaluation criterion in solid tumors; HI, hyperintensity percentage of tumor; CR, complete response; PR, partial response; SD, stable disease; PD, progressive disease; WI, weighted imaging.
